# A history of traumatic brain injury is associated with poorer cognition and imaging evidence of altered white matter tract integrity in UK Biobank (*n* = 50 376)

**DOI:** 10.1093/braincomms/fcae363

**Published:** 2024-10-11

**Authors:** Donald M Lyall, Emma R Russell, Joey Ward, William Stewart

**Affiliations:** School of Health and Wellbeing, University of Glasgow, Glasgow G12 8TB, Scotland, UK; School of Psychology and Neuroscience, University of Glasgow, Glasgow G12 8QB, Scotland, UK; School of Health and Wellbeing, University of Glasgow, Glasgow G12 8TB, Scotland, UK; School of Psychology and Neuroscience, University of Glasgow, Glasgow G12 8QB, Scotland, UK; Department of Neuropathology, Queen Elizabeth University Hospital, Glasgow G51 4TF, Scotland, UK

**Keywords:** traumatic brain injury, neurodegenerative disease, UK Biobank, diffusion tensor imaging, white matter

## Abstract

Traumatic brain injury (TBI) is a risk factor for neurodegenerative disease. We currently have no means to identify patients most at risk of neurodegenerative disease following injury and, resultantly, no means to target risk mitigation interventions. To address this, we explored the association between history of traumatic brain injury with cognitive performance and imaging measures of white matter integrity. From the UK Biobank imaging sub-study (*n* = 50 376), participants were identified with either self-reported (*n* = 177) or health record coded broad- (injury codes; *n* = 1096) or narrow-band (TBI specific codes; *n* = 274) TBI, or as controls with no such documented history (*n* = 49 280). Cognitive scores and imaging measures of corpus callosum white matter integrity were compared between injury participants (versus no injury), corrected for age, sex, socioeconomic status and medications. TBI was associated with poorer cognitive and imaging phenotypes. The strongest deleterious associations were for narrow-band injury (*β* difference 0.2–0.3; *P* < 0.01). All cognitive and imaging phenotypes were strongly inter-correlated (*P* < 0.001). This study provides insight into possible early biomarkers predating neurodegenerative disease following brain injury. Measures of cognition and white matter following injury may provide means to identify individuals most at risk of neurodegenerative disease, to which mitigation strategies might be targeted.

## Teaser

Lyall *et al*. show using electronic health record and self-report data, that traumatic brain injury is associated with worse cognitive and white matter microstructural integrity in ~50 000 non-demented participants from UK Biobank. Such measures may provide means to identify individuals most at risk of neurodegenerative disease.

## Graphical Abstract

**Graphical abstract:**
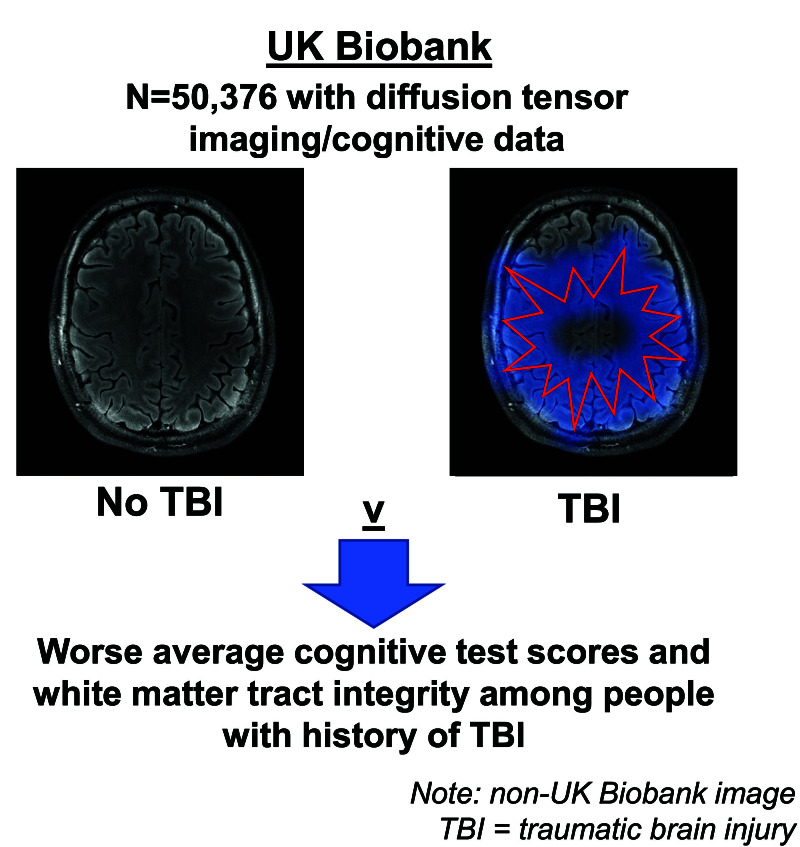

## Introduction

Approximately 885 000 people in the UK are living with dementia, with this number expected to rise to over 1.5 million by 2040, representing an estimated annual care cost of over £90 billion/year.^1^ Nevertheless, despite many decades of research, we still have no effective therapies for patients living with dementia and related neurodegenerative diseases (NDD). As such, identifying potential risk factors for disease and acting to reduce these is a priority.^2-6^ There is growing recognition that traumatic brain injury (TBI) is associated with increased risk of a range of NDD, with between 3 and 15% of dementia in the community attributable to TBI.^2,7^ Currently, however, we have no means to identify individuals most at risk of adverse brain health outcomes following TBI and, as a result, no means to target risk mitigation strategies.

It is now widely acknowledged that the pathological processes leading to NDD have onset many years before clinical diagnosis.^8,9^ For example, Alzheimer’s disease (AD) is considered to exist on a prodromal continuum where, prior to a dementia diagnosis, evidence of evolving, subclinical pathology might be detectable through tests of cognition and biomarkers, including brain imaging.^10,11^ In line with this, known risk factors for AD have been shown to associate with poorer cognitive performance and measurable brain imaging changes in individuals without dementia.^12^ Diffusion tensor MRI includes multiple highly sensitive metrics of WM integrity, which has previously been shown to decline with age, is influenced by risk factors for AD and is disrupted (on average) in people with a diagnosis of AD.^13^

Previous imaging studies in individuals with prior histories of TBI and no known NDD diagnosis have shown relatively widespread effects in measures of WM integrity. Cummins *et al*.^14^ for example using tract-based spatial statistics, showed significant differences specifically in the corpus callosum body, splenium and genu, bilateral anterior coronal radiata as well as right posterior thalamic radiation (*n* = 31 cases versus 21 controls, aged around 70 years). Wang *et al*.^15^ showed significantly worse WM tract integrity in 47 participants with mild TBI history, compared with no history, in all regions of interest, namely the inferior and superior cerebellar peduncle, pontine crossing tract (mean age 30 years, standard deviation [SD] = 6.8 years); Very fundamentally across a wide number of studies, the field has been commonly limited by relatively small sample sizes, variable population characteristics (e.g. combat veterans; professional athletes; clinical) and heterogeneous analytic approaches, including adjustment for covariates used.^16-21^ UK Biobank (UKB) is a relatively large, prospective, general population cohort of ∼0.5 million participants (at baseline). Following enrollment, a sub-study of ∼50 000 UKB participants have undergone MRI including together with concurrent cognitive assessment. This study aimed to explore the relationship between history of TBI and aspects of WM and cognitive health to test our hypothesis that history of TBI is associated with measures of reduced cognitive performance and white matter integrity.

## Materials and methods

### Participants and research measures

UKB is a large, prospective cohort of over 500 000 generally healthy, adults aged 40–70 years (mean = 56.5, SD = 8.1) at recruitment (2006–2010) and assessed at one of 22 centres. Participants completed a range of physical and psychological test batteries including largely on-screen components. The first MRI scan was in 2014 and is planned to continue until *n* = 100 000 are scanned^22^ in 1 of 4 assessment centres (Cheadle; Newcastle; Reading; Bristol). Details on these assessments are available in open-access protocols.^22,23^ For all variables, we removed participants who chose not to answer/did not know (<5%).

### Ethical approval

This secondary data analysis study was conducted under the generic approval from the NHS National Research Ethics Service (approval letter dated 17th June 2011, ref. 11/NW/0382). Written informed consent was obtained from all participants in the study (consent for research, by UKB). Analyses were completed using UKB project #17689 (PI Lyall).

### Head injury ascertainment

History of TBI was captured by three means: (i) via self-reported ‘head injury’ at time of scan; (ii) using a broad and inclusive list of 1798 relevant ICD-10 codes capturing data on head injury (broad-band TBI); and (iii) via capture of data on 51 ICD codes specific to TBI and associated terms (narrow-band TBI) (Supplementary Table 1). Primary and secondary ICD-10 diagnoses were based on hospital episode and admission (HES) admission NHS data, as detailed by the open-access protocol (https://biobank.ctsu.ox.ac.uk/crystal/crystal/docs/HospitalEpisodeStatistics.pdf), from May 1995 to October 2021. Participants who had a relevant broad-, or narrow-band ICD code at any date prior to scan were included as cases; a narrow-band case was necessarily also a broad-band case. The small number of participants who self-reported head injury, but also had an ICD-10-derived TBI, were included in both exposure groups.

### Cognitive assessments

Measurement and validation of cognitive phenotypes have been detailed previously.^24,25^ We used data from six tests: log pairs-matching (memory) errors; fluid intelligence (verbal-numeric reasoning); log reaction time (processing speed); and matrix completion, tower rearranging and Symbol digit completion (all executive functions).

UKB cognitive assessment is fundamentally in two categories: bespoke tests which were first used at baseline assessment and then repeated at imaging (Pairs-matching; Fluid intelligence; Reaction time), and separate tests first conducted in-person at imaging. These are relatively similar but not identical to more common tasks from other batteries e.g. Wechsler Adult Intelligence Scale-IV: Matrix completion, Tower rearranging and Symbol digit substitution. None of these tests have normative data per se, although it has previously been shown that the assessments are largely relatively stable in participants across average 5 years, and show strong inter-correlations including with more traditional cognitive tests .^26^ There is slight variation in cognitive assessment Ns due to variation in when tests were introduced to the scan visit protocol (e.g. Matrix pattern completion was first used in June 2017).

### Brain imaging

MR 3T image sequence, equipment, acquisition and processing details are available, open-access, on the UKB website in the form of protocol (http://biobank.ctsu.ox.ac.uk/crystal/refer.cgi?id=2367) and imaging documentation (http://biobank.ctsu.ox.ac.uk/crystal/refer.cgi?id=1977). Detailed methodologies are described in previous open-access reports.^22,27^ For imaging data, we used imaging derived phenotypes processed (including quality control) by UKB.^22^ Specifically, for the purposes of this study we accessed data on fractional anisotropy (FA), mean diffusivity (MD) and isotropic volume fraction (ISOVF) within the genu, corpus and splenium of the corpus callosum, with higher FA, lower MD and lower ISOVF reflecting better white matter tract integrity.

### Statistical analysis

We report differences between TBI versus not with standardized betas (*β*) on the per-SD scale, based on linear regressions for continuous outcomes, plus 95% confidence intervals. For reaction time, outliers ≥3.30 SDs above the mean (<1%) were excluded (this did not affect final results). We controlled for age at time of scan, sex, Townsend (deprivation) index, and self-reported medication for any cholesterol, insulin, blood pressure and/or exogenous hormones (specifically hormone replacement therapy).^24^ Stata v14 was used for analyses. We corrected for potential type-1 error by setting the significance threshold conservatively to *P* = 0.001.

## Results

### Demographic information

The average age at MRI was 64.37 (SD = 7.80) years, with 26 146 (51.9%) of 50 376 imaging participants female and 8365 (17%) self-reporting cardiometabolic or exogenous hormone medication. We specifically included participants who reported head injury at time of scan, or broad/narrow-band TBI based on ICD-10 codes prior to the scan date. Within this imaging cohort, 177 (0.4%) participants self-reported history for head injury, while 1096 (2.2%) and 274 (0.5%) were identified with either prior ICD-coded broad-band or narrow-band TBI respectively. Power analysis using G*Power 3.1 estimated 92% power to find an effect size of Cohen’s *D* = 0.2 (‘small’) at *P* < 0.05 based on the smallest sample size of narrow-band TBI when compared with no history. Of participants providing self-reported head injury history, 23 (12%) were captured under broad- and 12 (6%) under narrow-band TBI ICD-10 coding. The average interval between injury and MRI scanning was 9 years for broad-band TBI (SD = 6.11) and 10.05 years for narrow-band TBI (SD = 6.37). Demographic differences are displayed in Supplementary Table 2.

### Cognitive data

Regression estimates for cognitive analyses are shown in Table 1. Self-reported head injury was associated with worse Symbol digit scores alone (*β*=−0.268, *P* = 0.001) and no other tests. Broad-band TBI was associated with worse Symbol digit substitution scores (*β*=−0.101, *P* = 0.002) specifically. Narrow-band TBI was associated with worse Symbol digit substitution (*β*=−0.223, *P* = 0.001) and Matrix completion scores (*β*=−0.180, *P* = 0.014). There were no associations with Reaction time, Pairs-matching, Tower rearranging or Fluid intelligence scores (all *P* > 0.05). Note that the associations between broad-band TBI and Symbol digit substitution, and narrow-band TBI and Matrix Completion, were not significant at the more conservative *P* = 0.001 threshold. The associations between self-reported injury, and narrow-band TBI with Symbol digit Substitution, remained significant.

**Table 1:** Cognitive associations between head injury (self-report; broad-band; narrow-band ICD10 codes) compared with no history

Variable	Coefficient (*β*)	*P*	Lower 95% CI	Upper 95% CI	*N*	*r* ^2^
*Self-report head injury*						
Log reaction time	0.006	0.939	−0.140	0.152	44 041	0.114
Log pairs-matching errors	−0.012	0.880	0.164	0.140	44 760	0.022
Log tower rearranging errors	−0.073	0.425	−0.252	0.106	31 855	0.050
**Digit symbol score**	**−0**.**268**	**0**.**001**	**−0**.**432**	**−0**.**104**	**32 160**	**0**.**205**
Matrix completion score	−0.156	0.084	−0.333	0.021	32 143	0.070
Fluid intelligence score	−0.086	0.276	−0.241	0.069	43 904	0.019
*Broad-band TBI*						
Log reaction time	0.016	0.596	−0.044	0.077	44 041	0.114
Log pairs-matching errors	−0.022	0.490	−0.085	0.041	44 760	0.022
Log tower rearranging errors	−0.009	0.802	−0.080	0.062	31 855	0.050
**Digit symbol score**	**−0**.**101**	**0**.**002**	**−0**.**166**	**−0**.**037**	**32 160**	**0**.**205**
Matrix completion score	−0.047	0.191	−0.117	0.023	32 143	0.070
Fluid intelligence score	−0.023	0.485	−0.087	0.041	43 904	0.019
*Narrow-band TBI*						
Log reaction time	0.049	0.431	−0.073	0.171	44 041	0.114
Log pairs-matching errors	−0.021	0.744	−0.147	0.105	44 760	0.022
Log tower rearranging errors	−0.141	0.057	−0.287	0.004	31 855	0.050
**Digit symbol score**	**−0**.**223**	**0**.**001**	**−0**.**356**	**−0**.**09**	**32 160**	**0**.**205**
**Matrix completion score**	**−0**.**18**	**0**.**014**	**−0**.**324**	**−0**.**036**	**32 143**	**0**.**071**
Fluid intelligence score	−0.069	0.29	−0.197	0.059	43 904	0.019

95% CI, confidence intervals; SE, standard error.

Betas are on the per-SD scale. Associations corrected for: age, sex, Townsend index (deprivation) and self-reported medication history (cardiometabolic; exogenous hormones). Significant associations at *P* < 0.05 highlighted in bold.

### Imaging data

Associations between history of TBI history compared with not are shown in Table 2. Self-report history of head injury compared with no history, was associated with differences primarily in the body (ISOVF *β*=0.257, *P* = 0.001; MD *β*=0.217, *P* = 0.006; FA *β*=−0.211, *P* = 0.009) and genu of the corpus callosum (FA *β*=−0.166, *P* = 0.009). Broad-band ICD-10-based TBI compared with no history was associated with worse average scores on all imaging phenotypes (*β* range = 0.068 to [−]0.172, *P* range <0.001 to 0.008). Narrow-band TBI compared with no history, was similarly associated with all imaging phenotypes but with generally larger magnitudes of effect (*β* range = 0.135 to [−]0.377, *P* range <0.001 to 0.046). Note that several associations, between self-report injury and body (MD; FA); broad-band TBI and splenium (FA), and narrow-band TBI and splenium/genu (ISOVF) were not significant at the more conservative *P* = 0.001 threshold.

**Table 2:** Imaging associations between head injury (self-report; broad-band; narrow-band ICD10 codes) compared with no history

Variable	Coefficient (*β*)	*P*	Lower 95% CI	Upper 95% CI	*N*	*r* ^2^
*Self-report head injury*						
Splenium (ISOVF)	0.147	0.076	−0.015	0.309	40 190	0.044
**Body (ISOVF)**	**0**.**257**	**0**.**001**	**0**.**107**	**0**.**407**	**40 190**	**0**.**186**
Genu (ISOVF)	0.135	0.088	−0.020	0.290	40 190	0.130
Splenium (MD)	0.134	0.096	−0.024	0.292	40 192	0.097
**Body (MD)**	**0**.**217**	**0**.**006**	**0**.**063**	**0**.**371**	**40 192**	**0**.**141**
Genu (MD)	0.143	0.061	−0.007	0.293	40 192	0.181
Splenium (FA)	−0.103	0.220	−0.268	0.062	40 192	0.013
**Body (FA)**	**−0**.**211**	**0**.**009**	**−0**.**369**	**−0**.**053**	**40 192**	**0**.**092**
**Genu (FA)**	**−0**.**166**	**0**.**036**	**−0**.**321**	**−0**.**011**	**40 192**	**0**.**125**
*Broad-band TBI*						
**Splenium (ISOVF)**	**0**.**068**	**0**.**045**	**0**.**002**	**0**.**135**	**40 190**	**0**.**044**
**Body (ISOVF)**	**0**.**154**	**<0**.**001**	**0**.**092**	**0**.**216**	**40 190**	**0**.**187**
**Genu (ISOVF)**	**0**.**106**	**0**.**001**	**0**.**043**	**0**.**170**	**40 190**	**0**.**130**
**Splenium (MD)**	**0**.**114**	**0**.**001**	**0**.**049**	**0**.**179**	**40 192**	**0**.**097**
**Body (MD)**	**0**.**165**	**<0**.**001**	**0**.**101**	**0**.**228**	**40 192**	**0**.**142**
**Genu (MD)**	**0**.**132**	**<0**.**001**	**0**.**071**	**0**.**194**	**40 192**	**0**.**182**
**Splenium (FA)**	**−0**.**134**	**<0**.**001**	**−0**.**202**	**−0**.**066**	**40 192**	**0**.**014**
**Body (FA)**	**−0**.**172**	**<0**.**001**	**−0**.**237**	**−0**.**107**	**40 192**	**0**.**093**
**Genu (FA)**	**−0**.**158**	**<0**.**001**	**−0**.**222**	**−0**.**094**	**40 192**	**0**.**125**
*Narrow-band TBI*						
**Splenium (ISOVF)**	**0**.**135**	**0**.**046**	**0**.**003**	**0**.**268**	**40 190**	**0**.**044**
**Body (ISOVF)**	**0**.**199**	**0**.**001**	**0**.**077**	**0**.**322**	**40 190**	**0**.**186**
**Genu (ISOVF)**	**0**.**175**	**0**.**007**	**0**.**048**	**0**.**301**	**40 190**	**0**.**13**
**Splenium (MD)**	**0**.**247**	**<0**.**001**	**0**.**118**	**0**.**376**	**40 192**	**0**.**097**
**Body (MD)**	**0**.**29**	**<0**.**001**	**0**.**164**	**0**.**416**	**40 192**	**0**.**142**
**Genu (MD)**	**0**.**272**	**<0**.**001**	**0**.**149**	**0**.**394**	**40 192**	**0**.**182**
**Splenium (FA)**	**−0**.**316**	**<0**.**001**	**−0**.**451**	**−0**.**181**	**40 192**	**0**.**014**
**Body (FA)**	**−0**.**377**	**<0**.**001**	**−0**.**507**	**−0**.**248**	**40 192**	**0**.**093**
**Genu (FA)**	**−0**.**329**	**<0**.**001**	**−0**.**456**	**−0**.**202**	**40 192**	**0**.**125**

95% CI, 95% confidence intervals; FA, fractional anisotropy; ISOVF, isotropic volume fraction; MD, mean diffusivity; SE, standard error.

Betas are on the per-SD scale. Associations corrected for: age, sex, Townsend index (deprivation) and self-reported medication history (cardiometabolic; exogenous hormones). Significant associations at *P* < 0.05 highlighted in bold. Higher ISOVF, MD, pairs-matching errors and log rearranging errors = worse.

### Imaging/cognitive inter-correlations

Unadjusted Pearson *r* correlations showed consistent inter-correlations between imaging and cognitive phenotypes (range = 0.02 to 0.83, all *P* < 0.001; Supplementary Table 3).

### Additional analyses

There was little evidence of interactions between TBI and sex on any outcome (Supplementary Table 4). There were 5 significant interactions out of 45 tests at *P* < 0.05, e.g. broad-band TBI on genu MD (interaction β = 0.166, i.e. a 0.166 SD larger effect in males than females; *P* = 0.009), but no interactions were significant at the more conservative *P* = 0.001 threshold. Results were similar with univariate and/or less-adjusted models (e.g. simply age- and sex-adjusted).

## Discussion

Among this general population cohort aged around 61 years at assessment and with no known history of NDD, compared with participants with no history of TBI, after correction for multiple testing, participants with a history of TBI demonstrated poorer performance in the Symbol digit test (of executive function, working memory and information processing speed), and showed imaging evidence of significantly worse white matter integrity. These findings were broadly consistent across multiple methods of injury ascertainment, ranging self-reported history of head injury to TBI-specific ICD code outcomes captured from electronic health records. The effect size was dependent on means of TBI identification, with the effect size smallest for self-identified (around *β* = 0.2 SDs) and largest for narrow-band TBI (*β* = 0.2–0.3 SDs). This is a significant contribution to understanding because much prior literature was based on relatively small sample sizes including from varying populations (e.g. athletes; conflict veterans) and/or with relatively short follow-up since injury. This study demonstrates significantly lower average cognitive test scores (Symbol digit substitution) and brain WM tract integrity in a relatively large, (somewhat) older age, general population cohort, on average 9–10 years after injury.

A small proportion of associations and interactions with sex were nominally significant at *P* < 0.05 but not the more conservative 0.001 threshold; these may reflect a degree of type-1 error. What survived correction was primarily self-report and narrow-band TBI on Symbol digit scores and most broad- and narrow-band TBI associations with imaging. This cognitive test is purported to reflect a degree of information processing speed, visuospatial ability and fluid cognitive ability.^28^ There was limited evidence of sex-specific interactions although larger (sex-specific) sample sizes may be necessary to effectively power such tests. Finally, when considering imaging and cognitive measures together, these showed inter-correlation, with performance in cognitive testing directly correlated with MRI measures of white matter integrity. We observed consistent, medium-to-strong associations between cognitive and DTI phenotypes here. This is consistent with a general factor (aka ‘*g*’) of brain health where cognitive and structural brain phenotypes correlate strongly due to shared genetic and non-genetic influences.^29^

There are implications to these findings: TBI is an established dementia risk factor^2^ and these results suggest a degree of poorer than average cognitive health (compared with no history of TBI), which may be part of the observed dementia prodrome.^30^ Fundamentally, the effect sizes reported were relatively small in magnitude and are unlikely to have immediate clinical implications, but suggest long-lasting impact on particularly WM commissural health which may be a biomarker for subsequent NDD risk. WM tract integrity is a well-established substrate of general cognitive ability,^31^ and commissural tracts may represent an imaging biomarker of vulnerability to subsequent decline. The different ascertainment methods (i.e. self-report versus ICD-10 based) had relatively little overlap but ultimately similar associations with cognitive and brain imaging outcomes. This may reflect some degree of participant self-report error. Rutter *et al*. observed relatively high frequencies of ‘error’ in around 50 000 UKB participants with longitudinal data (defined as changes in ostensibly stable or historic values).^32^ The low overlap between self-report versus ICD-10 head injury could reflect some of this, or that some individuals did not seek medical attention, including potentially not-at-random. It is possible that the different magnitudes of effect/association between self-reported narrow-/broad-band TBI may reflect differences in severity of TBI.

### Limitations and future research

The current study has limitations. UKB has relatively established ‘healthy bias’ which is particularly pronounced in the imaging sub-sample and may adversely influence exposure/outcome association estimates.^33^ While this study investigated TBI in 3 ways (self-report; broad-band and narrow-band based on ICD codes), detailed TBI-related information, e.g. Glasgow Coma Scale scores, were not available. The cognitive battery is relatively brief compared with more comprehensive assessments, and there are some test-specific limitations (e.g. floor effects in the memory test).^25^ The cognitive test scores do not have normative values, i.e. absolute scores that reflect cognitive impairment per se. The cognitive tests often have relatively narrow range. For example, the memory test has a relatively high frequency of zero-inflation (i.e. no errors), and the fluid intelligence test ranges from 0 to 12, which may variously lead to ceiling and floor effects.^25^ There may be instances of undiagnosed cognitive impairment (e.g. dementia), and/or participants may have scored poorly for reasons other than cognitive health (e.g. physical frailty in the reaction time tests).

The TBI data is based on electronic health record data which do not consistently specify severity, and only state the first instance of an ICD code; this means that there is probably variation in TBI severity which the current data do not characterize well. The self-reported head injury phenotype is to some extent open to interpretation and could reflect a variety of incidents and/or pathologies. There is a relatively low frequency of ICD 10-defined TBI in this cohort, which could reflect that HES-based ascertainment underestimates injury in the general population. Some TBIs may go un-reported and/or be in primary care diagnoses which were not analysed here.

Future studies may investigate differences in brain structural phenotypes beyond WM tractography, for example, subcortical volumes, metrics of cerebrovascular health and/or longitudinal change in participants with multiple imaging assessments.

### Conclusion

Historic TBI, based on self-report or formal diagnosis, is to varying extents associated with differences in cognitive health (specifically Symbol digit substitution scores) and white matter integrity in individuals with no known diagnosis of NDD. These differences could be important insights into pre-dementia pathology and suggest potential routes to early intervention and targeting.

## Supplementary Material

fcae363_Supplementary_Data

## Data Availability

UKB is an open access resource available to verified researchers upon application (http://www.ukbiobank.ac.uk/). Analysis syntax is available on the Open Science Framework: https://osf.io/dj98e/.
